# A Clinical Index to Predict Progression from Mild Cognitive Impairment to Dementia Due to Alzheimer's Disease

**DOI:** 10.1371/journal.pone.0113535

**Published:** 2014-12-08

**Authors:** Sei J. Lee, Christine S. Ritchie, Kristine Yaffe, Irena Stijacic Cenzer, Deborah E. Barnes

**Affiliations:** 1 Division of Geriatrics, University of California San Francisco, San Francisco, California, United States of America; 2 San Francisco Veterans Affairs Medical Center, San Francisco, California, United States of America; 3 Jewish Home of San Francisco, San Francisco, California, United States of America; 4 Department of Psychiatry, University of California San Francisco, San Francisco, California, United States of America; 5 Department of Neurology, University of California San Francisco, San Francisco, California, United States of America; 6 Department of Epidemiology & Biostatistics, University of California San Francisco, San Francisco, California, United States of America; University of Leipzig, Germany

## Abstract

**Background:**

Mild cognitive impairment is often a precursor to dementia due to Alzheimer's disease, but many patients with mild cognitive impairment never develop dementia. New diagnostic criteria may lead to more patients receiving a diagnosis of mild cognitive impairment.

**Objective:**

To develop a prediction index for the 3-year risk of progression from mild cognitive impairment to dementia relying only on information that can be readily obtained in most clinical settings.

**Design and Participants:**

382 participants diagnosed with amnestic mild cognitive impairment enrolled in the Alzheimer's Disease Neuroimaging Initiative (ADNI), a multi-site, longitudinal, observational study.

**Main Predictors Measures:**

Demographics, comorbid conditions, caregiver report of participant symptoms and function, and participant performance on individual items from basic neuropsychological scales.

**Main Outcome Measure:**

Progression to probable Alzheimer's disease.

**Key Results:**

Subjects had a mean (SD) age of 75 (7) years and 43% progressed to probable Alzheimer's disease within 3 years. Important independent predictors of progression included being female, resisting help, becoming upset when separated from caregiver, difficulty shopping alone, forgetting appointments, number of words recalled from a 10-word list, orientation and difficulty drawing a clock. The final point score could range from 0 to 16 (mean [SD]: 4.2 [2.9]). The optimism-corrected Harrell's c-statistic was 0.71(95% CI: 0.68–0.75). Fourteen percent of subjects with low risk scores (0–2 points, n = 124) converted to probable Alzheimer's disease over 3 years, compared to 51% of those with moderate risk scores (3–8 points, n = 223) and 91% of those with high risk scores (9–16 points, n = 35).

**Conclusions:**

An index using factors that can be obtained in most clinical settings can predict progression from amnestic mild cognitive impairment to probable Alzheimer's disease and may help clinicians differentiate between mild cognitive impairment patients at low vs. high risk of progression.

## Introduction

Mild cognitive impairment (MCI) is a common disorder, affecting 3–5% of adults over 60 and 15% of adults over 75 [1]. It is characterized by a decline in cognitive function that falls between the changes associated with typical aging and those fulfilling the criteria for dementia. MCI is commonly classified into two subtypes: amnestic MCI, which refers to clinically significant memory impairment that does not meet dementia criteria, and non-amnestic MCI, which refers to decline in other aspects of cognitive function such as attention, language, visuospatial function or executive function; in addition, MCI may be present in a single domain or may affect multiple domains [2], [3]. Although MCI was initially conceptualized as a transitional stage between normal cognitive aging and dementia, particularly Alzheimer's disease (AD), community-based studies suggest that many individuals diagnosed with MCI do not progress to AD at an accelerated rate and may even revert to normal [4]–[6]. However, receiving a diagnosis of MCI can be traumatic for patients and family members [7]. Therefore, it is critically important for clinicians who oversee their care to be able to provide them with information regarding their likelihood of progression to AD.

This issue is likely to be compounded over the coming years as a result of the current push to diagnose AD earlier in the course of the disease [8]–[10]. There is growing awareness that the neuropathological processes that underlie AD begin many years and even decades prior to the onset of symptoms [11]. Given the lack of disease-modifying treatments for AD and the recent failure of several promising new agents in clinical trials, it is believed that treatments may need to be initiated prior to the onset of symptoms to be effective. The National Institute on Aging and the Alzheimer's Association have recently proposed new diagnostic criteria for preclinical AD [8] and MCI due to AD [10] to facilitate earlier diagnosis, and it is likely that this will lead to an increase in the number of patients diagnosed with MCI.

The Alzheimer's Disease Neuroimaging Initiative (ADNI) is one of several international efforts to identify neuroimaging factors or biomarkers that can predict which individuals with MCI will progress to dementia and which individuals will not [12]. However, to date, no single neuroimaging factor or biomarker has emerged that can accurately predict progression. For example, Pittsburgh compound B appears to be sensitive but not specific in identifying patients with MCI who will progress to dementia [13]. In addition, many of the neuroimaging and biomarkers being studied would be impractical in most clinical settings because they either require special equipment or are costly or difficult to obtain.

We have recently developed a multi-domain risk prediction index that incorporates a combination of structural magnetic resonance imaging measures, functional dependence and cognitive test scores to stratify older individuals with amnestic MCI into those with a high versus low risk of converting to AD [14]. The objective of our current study was to develop a simplified, clinical risk prediction index that relies on items that can be measured relatively easily in most clinical settings and to compare its predictive accuracy with our more comprehensive index and with other available prediction tools.

## Methods

### Study population

Subjects were participants in the Alzheimer's Disease Neuroimaging Initiative 1 (ADNI-1), an ongoing, multicenter study initiated in 2003 to develop clinical, imaging, genetic and biochemical biomarkers for the early detection and tracking of AD [12], [15]. ADNI was designed to enroll 200 volunteers who were cognitively normal, 400 with amnestic MCI and 200 with AD. Detailed information on ADNI study procedures can be found at http://www.adni-info.org/Scientists/ADNIStudyProcedures.aspx. Data are publically available at http://adni.loni.ucla.edu/and were downloaded for this study on July 31, 2012.

This study focuses on the 382 ADNI participants who were diagnosed with amnestic MCI at baseline (95% single-domain, 5% multi-domain), had at least one follow-up visit and did not have any critical missing data. All subjects in ADNI were age 55–90 and had no evidence of cerebrovascular disease (Modified Hachinski Ischaemia Score ≤4) [16], no evidence of depression (Geriatric Depression Scale <6) [17], stable medications, a caregiver or informant, adequate vision and hearing to perform study procedures, good general health, a minimum of six grades of education or work equivalent, English or Spanish language fluency, and no medical contraindications to magnetic resonance imaging.

ADNI used standard, well-defined criteria to diagnose amnestic MCI and AD at all time points. Subjects with amnestic MCI met the following criteria: memory complaint verified by caregiver or informant, abnormal memory function based on education-adjusted cut-off on the Logical Memory II subscale (delayed paragraph recall) from the Wechsler Memory Scale – Revised [18], Mini-Mental State Examination [19] score of 24–30 (inclusive), Clinical Dementia Rating [20] score of 0.5, and cognitive and functional impairment not severe enough to meet criteria for AD or dementia. AD was defined based on the National Institute of Neurological and Communicative Disorders and Stroke and the Alzheimer's Disease and Related Disorders Association (NINCDS-ADRDA) criteria for probable dementia due to AD [21]. Therefore, the key differences between the amnestic MCI and AD diagnostic categories were: 1) AD required evidence of cognitive impairment in at least two domains including memory whereas MCI required impairment in memory (with or without another domain) and 2) AD required that cognitive impairment be severe enough to interfere with the ability to perform usual activities whereas MCI required that general cognition and functional performance be sufficiently preserved such that a diagnosis of AD could not be made. Thus, participants with MCI may have had some minor functional deficits at baseline, but these deficits were not severe enough to satisfy criteria for AD. Potential subjects who were taking anti-depressants with significant anti-cholinergic effects, neuroleptics, anxiolytics or sedative hypnotics were excluded [22].

All ADNI subjects or their proxies provided written, informed consent. This project was submitted for review to the UCSF Committee on Human Research (CHR). However, since it involved no contact with human subjects and utilized completely de-identified data, UCSF CHR determined this project did not require review.

### Measures

A restricted range of potential predictor variables was considered, focusing on items that were available in the ADNI dataset that could be assessed in a typical clinical setting at minimal cost and without special equipment. Therefore, we did not consider magnetic resonance imaging, positron emission tomography imaging, genetic factors or blood-based or cerebrospinal fluid biomarkers. All potential predictor variables were collected at either the screening or baseline visit. Potential predictors were examined in domains as described below.

Demographic variables included age, sex, race/ethnicity, education, marital status, and family history of AD.

Medical history was determined based on self-report using a comprehensive list of conditions and categorized for the current study as history of hypertension, other cardiovascular disease (e.g., high cholesterol, coronary artery disease), diabetes, respiratory conditions (e.g., asthma, pneumonia), hematopoetic/lymphatic or malignancy (e.g., anemia, prostate cancer), kidney disease (e.g., kidney stones, renal insufficiency), smoking, head injury, and thyroid conditions (e.g., hypothyroidism, hyperthyroidism).

Symptoms and vital signs included low energy or insomnia (self-report), abnormal gait (neurologic assessment), blood pressure (normal: diastolic <90 mmHg and systolic <140 mmHg; stage 1 hypertension: diastolic 90–99 or systolic 140–159; stage 2 hypertension: diastolic ≥100 or systolic ≥160), pulse (beats/minute) and body mass index (BMI, kg/m^2^).

Functional dependence was assessed with individual items from the 10-item Functional Assessment Questionnaire in which caregivers were asked about the participant's level of functional dependence with questions focused on instrumental activities of daily living such as paying bills and shopping [23].

Neuropsychiatric symptoms were assessed with individual items from the 12-item Neuropsychiatric Inventory in which caregivers were asked about the frequency and severity of behaviors such as stubbornness and impulsivity [24].

Cognitive function was assessed with individual items from the Alzheimer's Disease Assessment Scale – cognitive subscale [25]. Specific items were considered as potential predictors for the current study if they could be reasonably performed in a typical clinical setting and included: recall of a 10-word list (average over 3 learning trials); ability to perform a series of 5 increasingly complex commands (e.g., make a fist; tap each shoulder twice with two fingers while keeping your eyes shut); ideational praxis (ability to correctly address an envelope); and orientation to time and place. In addition, Category Fluency [26] was assessed by asking participants to name as many different animals as possible in one minute, and then as many vegetables as possible in one minute. The Clock Drawing Test [27] was utilized to assess visuospatial function, with scores ranging from 0 to 5 and lower scores reflecting greater impairment. Other cognitive measures that were collected as part of ADNI were not considered as potential predictors in our risk index because they were either proprietary or were considered too time-intensive for the clinical setting.

### Progression to Dementia due to AD

Our primary outcome was progression to probable dementia due to AD. As part of ADNI-1, subjects were reassessed at 6, 12, 18, 24 and 36 months. Additional follow-ups are being performed annually as part of ADNI-2 [15]. Potential progressions are reviewed by a clinical monitor and confirmed by the progression committee to establish a consensus diagnosis. Diagnoses are based on National Institute of Neurological and Communicative Disorders and Stroke and the Alzheimer's Disease and Related Disorders Association (NINCDS-ADRDA) criteria for probable dementia due to AD [21]. Since the exact date of progression to dementia was not known, we used the midpoint between the last follow-up without a dementia diagnosis and the first follow-up with a dementia diagnosis. Subjects that didn't progress were censored at the time of their last interview.

### Statistical analyses

We first examined univariate distributions of all potential predictors to assess for evidence of outlier values. Bivariate associations between potential predictors and the outcome (conversion to AD) were then examined using t-tests or analysis of variance for continuous variables and Chi-square tests for categorical variables. Clinically meaningful categories were utilized when available (e.g., blood pressure, BMI); otherwise, quartiles were utilized. Variables with ≤5 subjects in a given cell were not considered.

To account for differential length of follow-up and attrition, Cox proportional hazards regression analyses were utilized to identify factors associated with time to AD. We first performed a series of domain-specific Cox analyses in which we identified the variables from within each domain that were associated with conversion to AD at p<0.20. A less stringent p-value was used at this step to ensure consideration of a wide range of potential predictors. Variables identified from within each domain were then considered together in a single Cox regression analyses, and those variables that were significantly associated with time to AD at p<0.05 were retained in the final model. Each variable in the final model was then assigned a point value by dividing its model coefficient value by the absolute value of the smallest coefficient in the model and rounding to the nearest integer.

Model discrimination was assessed using Harrell's c statistic [28]. Bootstrapping methods were used to validate the final model by estimating a correction value for optimism due to overfitting. [29] Model calibration was assessed by plotting the observed vs. predicted progression rates (based on Kaplan-Meier estimates) at 3 years as a function of point scores. Finally, the prognostic accuracy of the current clinical index was compared with our previously developed full index using bootstrapping methods and by plotting observed vs. predicted progression rates as a function of point scores for the two indices.

STATA 12.1 (College Station, TX) and SAS 9.2 (Cary, NC) were used for statistical analysis.

## Results

Baseline characteristics of the study population are shown in Table 1. Subjects had a mean (SD) age of 75 (7) years; 36% were women, 91% were non-Hispanic white, 20% had ≤12 years of education and 80% were married. Comorbid medical conditions were common, with approximately half having a history of hypertension or other cardiovascular disease. Caregivers reported that nearly 1 in 5 participants were stubborn and resistive to help from others; became upset when separated from them or had difficulty remembering important events such as appointments and holidays whereas fewer participants (6%) had difficulty shopping alone. Approximately 1 in 5 participants had impaired clock test scores. A total of 179 (46.9%) study participants converted to probable AD over a mean (SD, range) follow-up period of 2.9 (1.1, 0.5–4.0) years. Of the 203 who did not progress, 71 had <3 years of follow-up data and were censored while 132 were followed for at least 3 years.

**Table 1:** Baseline Characteristics of 382 Participants with Amnestic Mild Cognitive Impairment (MCI).

Characteristic	No. (%) or Mean (SD)
Demographic factors	
Age, years	75±7
Gender, female	137 (36)
Race, non-Hispanic white	346 (91)
Education, ≤12 years	77 (20)
Marital status, married	307 (80)
Family history of AD	132 (35)
Selected medical conditions	
Hypertension	188 (49)
Other cardiovascular disease	207 (54)
Diabetes	32 (8)
Current Smoker	13 (3)
Selected symptoms and vital signs	
Low energy	78 (20)
Insomnia	46 (12)
Abnormal gait	35 (9)
Systolic blood pressure, mmHg	132.3 (19.4)
Pulse, beats/minute	66±10.7
Body mass index <22 kg/m^2^	321 (84)
Selected functional (FAQ) items	
Difficulty shopping alone for household items	22 (6)
Difficulty remembering appointments and events	81 (21)
Difficulty traveling alone outside neighborhood	39 (10)
Difficulty understanding TV/books	16 (4)
Selected neuropsychiatric (NPI) items	
Stubborn and resistive to help from others	71 (19)
Becomes upset when separated	68 (18)
Feels too good or acts excessively happy	11 (3)
Selected cognitive items	
ADAS-cog – verbal recall over 3 trials, no. words	5.42±1.40
ADAS-cog – orientation, no. correct	7.36±0.94
Category fluency – vegetables, no. correct	10.8±3.5
Clock Drawing Test, score <4	79 (21)

Table includes selected demographic and medical history variables and all items from within each domain that were associated with conversion to AD (p<0.20). AD, Alzheimer's disease; ADAS-Cog, Alzheimer's Disease Assessment Scale – cognitive subscale; FAQ, Functional Assessment Questionnaire; NPI, Neuropsychiatric Inventory; SD, standard deviation. Data missing as follows: Blood pressure (4), pulse (1), FAQ (3).

We next determined which factors were associated with progression to dementia due to AD within each risk domain (p<0.20). Demographic predictors included being female or married. None of the medical history variables considered were associated with progression to AD. In the symptoms and vital signs domain, low energy, insomnia, abnormal gait, high blood pressure, high pulse and low BMI (<22) were all associated with increased risk of AD. Within the functional domain, caregiver report that a participant had difficulty shopping alone for household items, remembering important appointments and events, or traveling out of the neighborhood were associated with increased risk of AD while difficulty paying attention to and understanding TV or books was associated with a lower risk. Of the neuropsychiatric symptoms considered, caregiver reports that a participant was stubborn and resistive to help from others or became upset when separated from them were associated with increased dementia risk while feeling good or happy were associated with reduced risk. Finally, of the cognitive measures considered, poor scores on the word recall and orientation on the ADAS-cog, category fluency for vegetables, and the clock test all were associated with increased dementia risk.

The factors that emerged as being independently predictive of conversion from amnestic MCI to dementia due to AD and were retained in the final model are shown in Table 2 along with their hazard ratios and number of points assigned. Key predictors included being female (1 point); caregiver report that a participant was stubborn/resistant to help (2 points), became upset when separated from them (1 point), or had difficulty shopping alone for household items (2 points) or remembering important appointments and events (2 points); and poor performance on individual neuropsychological test items including 10-word recall (0 to 4 points), orientation (0 to 2 points) or Clock Test (2 points) (see S1 Appendix).

**Table 2:** Factors Associated with Conversion from Amnestic MCI to AD*.

Characteristic	KM Estimated 3-Year Conversion	Hazard Ratio(95% CI)	Points
Demographic			
Gender			
Male	45.1	1	
Female	53.5	1.7 (1.2, 2.3)	1
Functional dependence			
Difficulty shopping alone			
No	46.0	1	
Yes	80.7	2.4 (1.4, 4.1)	2
Difficulty remembering appointments			
No	40.0	1	
Yes	78.3	2.0 (1.4, 2.8)	2
Neuropsychiatric symptoms			
Stubborn/resistive to help			
No	43.1	1	
Yes	68.8	1.8 (1.3, 2.6)	2
Upset when separated			
No	45.5	1	
Yes	61.8	1.4 (1.0, 2.1)	1
Cognitive measures			
ADAS-cog, mean number words recalled (3 trials of 10 words)			
>6	28.1	1	
5.1–6	38.2	1.6 (1.0, 2.6)	1
4.1–5	67.2	2.9 (1.8, 4.4)	3
≤4	71.1	4.4 (2.8, 6.9)	4
ADAS-cog, orientation (no. correct of 8)			
8	36.7	1	
7	60.2	1.6 (1.1, 2.3)	1
≤6	72.4	1.9 (1.2, 2.8)	2
Clock Test score (no. correct of 5)			
4–5	42.2	1	
0–3	71.0	1.8 (1.3, 2.6)	2

AD, Alzheimer's disease; ADAS-cog, Alzheimer's Disease Assessment Scale – cognitive subscale; CI, confidence interval; MCI, mild cognitive impairment.

*Only factors retained in the final model are included.

**Figure 1:**
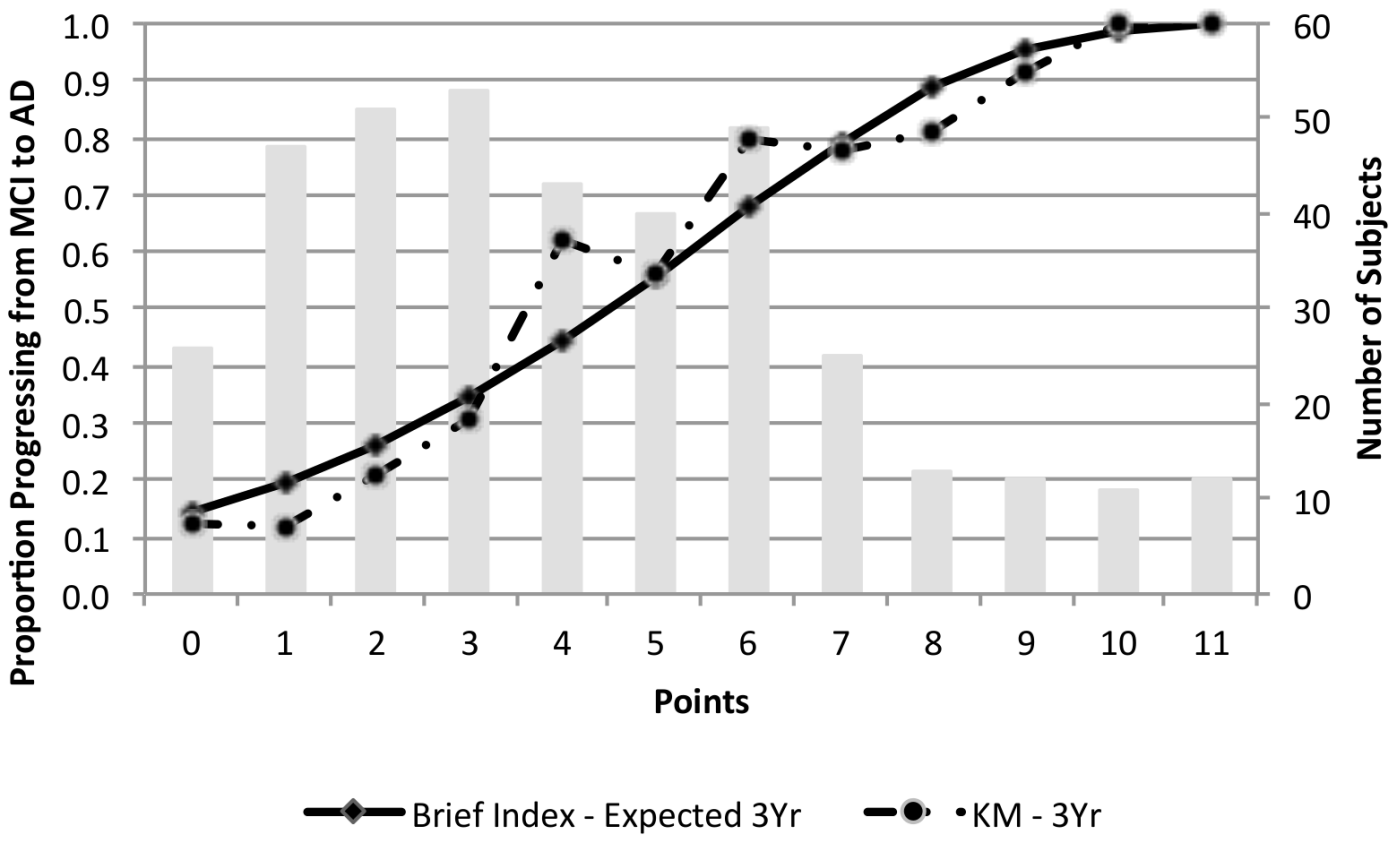
Observed versus Predicted Conversion from Amnestic MCI to AD over 3 Years by Brief Clinical Index Point Score. The solid line shows the proportion of subjects predicted to progress from amnestic mild cognitive impairment (MCI) to probable Alzheimer's disease (AD) over three years as function of their brief clinical index point score, while the dotted line shows the actual proportions that progressed at each point score value based on three-year Kaplan-Meier (KM) estimates. The vertical bars show the number of individuals at each point score value (right vertical axis).

Points from each item in the index were summed to create a total point score, which could range from 0 to 16 and had a mean (SD) of 4.2 (2.9). The point score had good accuracy for prediction of conversion from amnestic MCI to AD (Harrell's c, 0.75; 95% CI: 0.72, 0.79). Validation using boot-strapping techniques estimated our optimism as 0.04, resulting in an optimism-corrected Harrell's c-statistic of 0.71. Fig. 1 shows good concordance between the observed and expected rates of progression to dementia at 3 years as a function of point scores, suggesting good calibration for the index.

**Figure 2:**
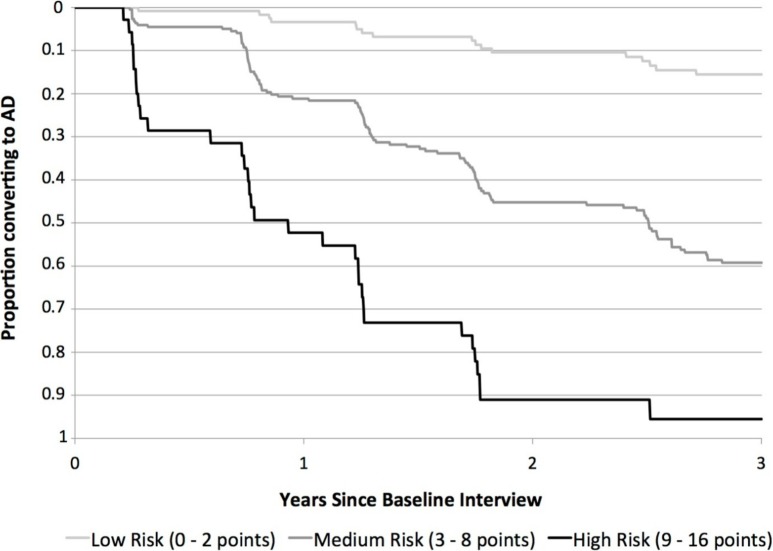
Proportion of Subjects with Amnestic MCI Who Converted to AD in Low, Medium and High Risk Groups. Fourteen percent of subjects with low risk scores (upper line, 0–2 points, n = 124) progressed from amnestic MCI to AD over three years compared to 51% of those with moderate risk scores (middle line, 3–8 points, n = 223) and 91% of those with high risk scores (lower line, 9–16 points, n = 35).

The index also was able to stratify participants into low, moderate and high risk groups for progression to dementia within 3 years. When subjects were grouped based on their risk scores, 14% of subjects with low risk scores (0 to 2 points, n = 124) progressed to dementia over 3 years, compared to 51% of those with moderate risk scores (3 to 8 points, n = 223) and 91% of those with high risk scores (9 to 16 points, n = 35) (Fig. 2).

**Figure 3:**
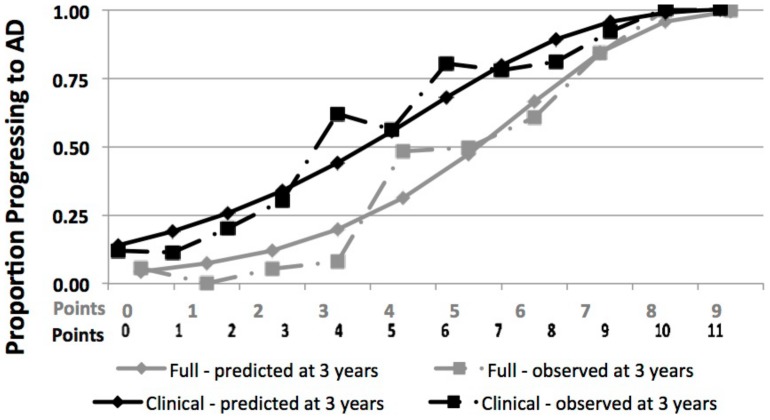
Comparison of the Full and Brief Clinical AD Prediction Indexes. The predicted (solid lines) and observed (dashed lines) are shown as a function of risk score values based on the previously published full index (light grey lines) and brief clinical index (dark grey lines). Prognostic accuracy was significantly higher for the full index (optimism corrected Harrell's c, 0.74) than the brief clinical index (0.71). However, the plot shows good concordance between observed and predicted risk for both indices.

The accuracy of the clinical index was significantly lower than our full index, which incorporated neuroimaging measures and scores from full functional questionnaires and neuropsychological tests (Harrell's c, 0.78; 95% CI: 0.75, 0.81; p = 0.02). The optimism-corrected Harrell's c for the full index was 0.74. However, the plot of observed vs. predicted progression rates for the two indices showed good concordance (Fig. 3).

## Discussion

We successfully developed a brief clinical index to predict whether older adults diagnosed with amnestic MCI will progress to probable AD within 3 years. Our index utilized 8 items that are readily obtainable in most clinical settings: gender; 4 questions regarding caregiver report of the patients' behaviors (stubborn/resists help and upset when separated) and functional status (difficulty shopping alone and forgets appointments) and 3 items focusing on ability to complete basic cognitive tasks (10-item list word recall, orientation to time and place and clock draw test). Our index had good discrimination with a Harrell's c-statistic of 0.75, which was only slightly lower (0.71) after correction for potential optimism.

Our prediction index also was able to successfully classify study participants into low, moderate and high risk groups. Nearly one-third of subjects were identified as having a <20% risk of progressing to AD, meaning these patients could be reassured that their risk of progression is low. Conversely, nearly 10% of subjects were identified as having a >90% risk of progressing to AD. Once identified, these high risk patients and their families could be counseled to prepare advance directives and support systems to allow for better care as cognitive impairment progressed. The moderate risk group could be followed closely for signs of progression to AD. By focusing on factors that could be readily obtained in most clinical settings, we believe our index could be useful for a wide range of providers to counsel patients diagnosed with amnestic MCI.

In both the clinical index and the full index, cognitive and functional measures were key predictors of progression from amnestic MCI to AD. These results are consistent with current theoretical models suggesting that cognitive and functional measures decline later in the disease trajectory, closer to the time of progression [11]. Thus, while neuroimaging and cerebrospinal fluid markers may be useful in identifying which cognitively normal patients will ultimately develop dementia, functional and neuropsychological markers may be more important in identifying MCI patients who are most likely to convert to dementia.

The accuracy of our brief index also compares favorably to other prognostic models that have examined progression from MCI to dementia using single or multiple predictors. Prior studies have found that c statistics for prediction of progression from MCI to AD typically range from 0.66 to 0.73 for studies that utilize structural MRI measures either alone or in combination with cerebrospinal fluid biomarkers [30]–[33]. One study found that a combination of MRI measures and cognitive test scores yielded a c statistic of 0.68 [34], although another study examining slightly different MRI and cognitive predictors yielded a higher c statistic of 0.80 [35]. Prognostic accuracy has generally been highest for models that include both cognitive and functional measures, with a relatively small gain in accuracy when neuroimaging measures or biomarkers are added [35], [36].

To our knowledge, our studies are the first to develop point scores to predict the risk of progression from amnestic MCI to AD. Point scores may be especially useful in clinical settings where they can be used to classify patients into low, moderate and high-risk groups, and guide patients and caregivers to appropriate next steps. The current study suggests that our brief index, which includes only measures that are relatively easy to obtain in most clinical settings, is only slightly less accurate than our full index, which includes structural MRI measures and full cognitive and functional scales (Harrell's c, 0.78 vs 0.75, p = 0.02). For most clinical settings, we believe that this modest decrease in predictive accuracy will be acceptable and outweighed by our brief index's ease-of-use.

Prediction indexes such as ours are becoming increasingly common in clinical practice. The Framingham cardiovascular risk calculator [37], the CHADS2 stroke risk calculator for atrial fibrillation [38] and the FRAX calculator for fracture risk in osteoporosis [39] are all examples of prediction indexes that are widely used in clinical practice. Our prediction index has similar predictive power (as measured by the Harrell's c-statistic) as these widely used indexes, suggesting that it may be useful in clinical practice.

Although there have been concerns that prediction indexes may harm patients by providing information that is worrisome, to our knowledge, no studies have documented actual harms. One study focusing on mortality prediction in older adults found that most patients wanted to discuss prognosis and life expectancy so they could “make the most of the life they had left.” [40] Thus, we believe that clinicians should ask patients whether they would like to discuss their chances of developing dementia. If the patient or family would like to know more, then a fuller discussion outlining the results of our predictive index may be helpful.

Our results should be interpreted in light of the study's strengths and limitations. Strengths include a rigorous, standardized methodology for determining the initial diagnosis of amnestic MCI as well as any potential progression to dementia. Second, the multi-site longitudinal cohort design of ADNI suggests that our results are more likely to be generalizable across different geographic settings.

Our study also has important limitations. First, although bootstrapping techniques allowed us to estimate the degree of overfitting and likely optimism in the Harrell's c statistic, external validation in a different cohort would provide additional assurance that our index is accurate in the general population of patients with amnestic MCI. Second, participants who volunteer for longitudinal studies such as ADNI may differ from patients who do not volunteer. Thus, our index may perform differently in community clinical settings. Third, our index requires information from both the patient as well as a caregiver, suggesting that our index cannot be used for patients without a reliable caregiver. Fourth, although we relied on factors which can be obtained in most clinical setting, further simplification of this index could ease its use in busy clinical settings. Fifth, it is possible that other cognitive tests such as cued recall tests may have similar accuracy to the ADAS-cog 3-trial recall test included in our index. Finally, ADNI was restricted to amnestic MCI; additional studies are needed to determine whether the accuracy of our index is similar in individuals with non-amnestic MCI.

In conclusion, we developed an 8-item point score to help providers predict progression from amnestic MCI to AD over 3 years. Our index showed good discrimination with an optimism-corrected Harrell's c-statistic of 0.71 and relies on information that should be relatively easy to obtain in most clinical settings. Our index may help stratify the growing population of older adults with amnestic MCI into those at low, intermediate and high risk of progressing to dementia due to AD.

## Supporting Information

S1 Appendix **MCI Risk Conversion Score Sheet.**
(DOCX)
