# Supplementary material for: A Clinical Index to Predict Progression from Mild Cognitive Impairment to Dementia Due to Alzheimer's Disease
Source: PLoS One. 2014 Dec 8;9(12):e113535. doi: 10.1371/journal.pone.0113535 (PMC4259326; doi:10.1371/journal.pone.0113535)
Supplement: S1 Appendix — MCI Risk Conversion Score Sheet. (DOCX) [file pone.0113535.s001.docx]

Appendix S1

| **MCI Risk Conversion Score Sheet** | **Points** | |
| --- | --- | --- |
| 1. Is your patient female? | No (0) | Yes (1) |
| 2) *Ask caregiver:* Is ________ stubborn and resistive to help from others? | No (0) | Yes (2) |
| 3) *Ask caregiver:* Does ________ become upset when separated from you? | No (0) | Yes (1) |
| 4) *Ask caregiver:* Does ________ have difficulty shopping alone for clothes, household necessities or groceries? | No (0) | Yes (2) |
| 5) *Ask caregiver:* Does ________ forget appointments, family occasions, holidays, or medications? | No (0) | Yes (2) |
| 6) What is the average number of words (out of 10) immediately recalled over 3 trials?^1^ | >6 (0)  >5 - 6 (1)  >4 - 5 (3)  ≤4 (4) | |
| 7) What is the patient’s orientation to person, time and place? (Number correct: name, month, date, year, day of week, season, place, time) ^2^ | 8 (0)  7 (1)  ≤6 (2) | |
| 8) Clock Test (Number correct: circular, numbers symmetrical, numbers correct, 2 hands, hands set to 11:10) ^3^ | 4-5 (0)  0-3 (2) | |
| Total point score (possible range: 0-16) |  | |

^1^Prepare cards with 10 high-frequency, high-imagery nouns printed in block letters on white cards. Hand the cards to the patient one at a time and ask them to read each word out loud and try to remember it. If they are unable to read, say the word for them and have them repeat it. After all 10 words have been read, ask them to tell you all the words they remember that were on the list. Record the number of words recalled. Perform this process three times and calculate the mean number of words recalled.

^2^Ask the patient the following questions: What is your name [must be exact, first and last]? What is the month [must be exact], What is today’s date [must be within 1 day]? What is the year [must be exact]? What day of the week is it [must be exact]? What season is it [must be within one week prior to onset or two weeks after termination]? What time is it [must be within one hour]? Where are we [partial names are acceptable; generic names such as ‘hospital’ or ‘doctor’s office’ are not]? Record the number of questions answered correctly.

^3^Ask the patient to draw the face of a clock showing the numbers and two hands set to ten after eleven. Award 1 point for each of the following: approximately circular face, symmetry of number placement, correctness of numbers, presence of two hands, shorter hand pointing toward 11 and longer hand pointing toward 2.
